# Molecular profiling of solid tumors by next-generation sequencing: an experience from a clinical laboratory

**DOI:** 10.3389/fonc.2023.1208244

**Published:** 2023-07-06

**Authors:** Pratibha Bhai, Jacob Turowec, Stephanie Santos, Jennifer Kerkhof, LeeAnne Pickard, Aidin Foroutan, Daniel Breadner, Matthew Cecchini, Michael A. Levy, Alan Stuart, Stephen Welch, Christopher Howlett, Hanxin Lin, Bekim Sadikovic

**Affiliations:** ^1^ Molecular Genetics Laboratory, London Health Sciences Centre, London, ON, Canada; ^2^ Verspeeten Clinical Genome Centre, London Health Sciences Centre, London, ON, Canada; ^3^ Department of Pathology and Laboratory Medicine, Schulich School of Medicine and Dentistry, Western University, London, ON, Canada; ^4^ Department of Oncology, Schulich School of Medicine and Dentistry, Western University, London, ON, Canada; ^5^ Molecular Genetics Laboratory, Alberta Precision Laboratories, Edmonton, AB, Canada

**Keywords:** solid tumor, NGS, lung cancer, colon cancer, melanoma

## Abstract

**Background:**

Personalized targeted therapies have transformed management of several solid tumors. Timely and accurate detection of clinically relevant genetic variants in tumor is central to the implementation of molecular targeted therapies. To facilitate precise molecular testing in solid tumors, targeted next-generation sequencing (NGS) assays have emerged as a valuable tool. In this study, we provide an overview of the technical validation, diagnostic yields, and spectrum of variants observed in 3,164 solid tumor samples that were tested as part of the standard clinical diagnostic assessment in an academic healthcare institution over a period of 2 years.

**Methods:**

The Ion Ampliseq™ Cancer Hotspot Panel v2 assay (ThermoFisher) that targets ~2,800 COSMIC mutations from 50 oncogenes and tumor suppressor genes was validated, and a total of 3,164 tumor DNA samples were tested in 2 years. A total of 500 tumor samples were tested by the comprehensive panel containing all the 50 genes. Other samples, including 1,375 lung cancer, 692 colon cancer, 462 melanoma, and 135 brain cancer, were tested by tumor-specific targeted subpanels including a few clinically actionable genes.

**Results:**

Of 3,164 patient samples, 2,016 (63.7%) tested positive for at least one clinically relevant variant. Of 500 samples tested by a comprehensive panel, 290 had a clinically relevant variant with TP53, KRAS, and PIK3CA being the most frequently mutated genes. The diagnostic yields in major tumor types were as follows: breast (58.4%), colorectal (77.6%), lung (60.4%), pancreatic (84.6%), endometrial (72.4%), ovary (57.1%), and thyroid (73.9%). Tumor-specific targeted subpanels also demonstrated high diagnostic yields: lung (69%), colon (61.2%), melanoma (69.7%), and brain (20.7%). Co-occurrence of mutations in more than one gene was frequently observed.

**Conclusions:**

The findings of our study demonstrate the feasibility of integrating an NGS-based gene panel screen as part of a standard diagnostic protocol for solid tumor assessment. High diagnostic rates enable significant clinical impact including improved diagnosis, prognosis, and clinical management in patients with solid tumors.

## Introduction

Molecular profiling of tumors has now become a routine and integral part of diagnosis, prognosis, and treatment planning for patients with advanced malignant cancers. The traditional molecular testing approach for targeted therapy decision-making involved testing a single hotspot mutation in a single patient at a time. However, recent sequencing technologies, i.e., next-generation sequencing (NGS), enabled simultaneous analysis of multiple genes in a single test across a large number of samples, while reducing the cost and turnaround time (TAT). Current oncology practice for patients with advanced stage tumors, e.g., non-small cell lung cancer (NSCLC), melanoma, and colorectal (CRC) adenocarcinoma, may involve treatment with molecularly targeted drugs or enrollment in clinical trials (1–3). The current guidelines for NSCLC recommend frontline comprehensive testing of all known actionable driver biomarkers including EGFR, ALK, ROS1, BRAF, KRAS, NTRK, MET, RET, HER2 [ERBB2], and PD-L1 with higher relevance in advanced disease stages, to choose the most appropriate targeted therapy for a patient (4, 5). PD-L1 expression status is evaluated in NSCLC tumors for eligibility of immune checkpoint inhibitors therapy (PD-1/PD-L1 inhibitors) (5). A comprehensive testing approach ensures a timely personalized treatment in NSCLC with maximum therapeutic efficacy and better patient outcomes. Targeted therapeutic approaches have successfully prolonged overall survival for CRC patients. RAS mutation status in metastatic colorectal cancer patients is central to the success of anti-EGFR targeted therapies. BRAF inhibitors and combination therapies have shown promising results in BRAF V600 mutated CRC patients. Most recent National Comprehensive Cancer Network (NCCN) guidelines recommend to evaluate metastatic CRC patients for RAS, mismatch repair, BRAF, HER2, VEGF, and PD-1/PD-L1 status to choose the appropriate initial and subsequent therapy (6). Melanoma development could be influenced by driver mutations such as BRAF, H/N/K-RAS, and C-KIT. Molecular testing for activating BRAF variants has become standard of care as recommended by NCCN and the European Society for Medical Oncology (ESMO) for stage III or IV melanoma, to evaluate the eligibility and efficacy of BRAF/MEK inhibitors targeting the BRAF-MEK-ERK pathway (7). KIT mutations in melanoma correlate with specific clinicopathological features and are candidates for KIT targeted therapies but has limited evidence of efficacy (8). Integration of these molecular markers in therapeutic decision-making highlights the utility of upfront molecular testing for the management of several tumor types.

Genetic information can also inform the diagnostic and prognostic aspects of the disease in individual patients. NRAS mutations found in 25% of melanoma cases have been associated with lower median overall survival and high aggressiveness with lack of efficient targeted therapies and also emerging resistance to existing treatments options (9). PIK3CA gene mutations are known as a good prognostic factor for breast carcinoma (10), and a poor prognostic marker for colorectal tumors (11). Also, association of TP53 mutations with unfavorable outcomes in many solid tumors including lung cancer (12) is not uncommon. IDH1/IDH2 mutations are associated with a favorable prognosis in patients with glioma and may confer a survival benefit for patients treated with radiation or alkylating chemotherapy [13, NCCN.org]. Loss of PTEN has been associated with favorable outcomes in endometrial cancers (14, 15).

Molecular profiling of tumors often provides diagnostic information like RB1 mutations in retinoblastoma and RET in thyroid tumors. Other examples include diagnostic utility of CTNNB1 mutations in pediatric desmid tumors (16) and IDH1/2 mutations in brain tumor subtypes (17). Recently, integration of molecular information for stratification and molecular subtyping of female genital tumors highlight the utility of molecular markers for tumor diagnosis and classification (18).

Implementation of standardized genomic screening protocols as part of routine clinical diagnostic workup and our expanding knowledge of genetic associations provide a growing impact on cancer patient management. Broad molecular profiling has now become essential for nearly all patients with metastatic solid tumors. Molecular profiling methodologies, guidelines, and practice standards are also subjected to continuous revision and updates as new technologies and markers become available. One of the major challenges other than continuous evolution of this field is the choice of disease-specific panels *vs*. more comprehensive panels as cost, TAT, and better utilization of resources in community settings play a huge role in making such decisions (19). Identification of patients who can significantly benefit from this powerful test, availability of timely results, and interpretation of findings have been a constant challenge in community settings.

Cancer Care Ontario (CCO) is an agency that formulates cancer diagnosis/treatment services-related guidelines for local healthcare professional to deliver best patient care in the province of Ontario Canada (https://www.cancercareontario.ca/). In Ontario, a province-wide standardized approach to molecular diagnostics has been adopted in recent years with initial implementation at London Health Science Centre (LHSC), which serves molecular testing on tumor samples of patients referred from Southwest Ontario, and all molecular tests were ordered based on an indication-based ordering system. In this study, we analyzed genetic test results from the first 3,164 tumor samples tested using a frontline NGS panel test at LHSC and evaluated the diagnostic utility of this panel in a clinical setting.

## Methods

### Targeted NGS panel

#### Patients/tumor samples

All tests were ordered based on an indication-based ordering system. Referring laboratories or local clinicians utilized a standardized form that had listed indications for CCO-approved testing (https://lhsc.omni-assistant.net/lab/Document/Handlers/FileStreamer.ashx?Df_Guid=61d249dd-e8c9-4bcb-9f36-c4a6246c6602&MostRecentDocument=true). All cases were then reviewed by a local pathologist and the approved molecular testing was ordered based on the diagnosis. The review included assessment of the pathology report and immunohistochemical testing was performed. In some cases, additional immunohistochemistry testing is performed by the local pathologist to confirm the diagnosis before initiating the indicated tested. Requests for testing outside of the approved CCO pathways were typically rejected or re-directed to other pathways. A total of 3,164 solid tumor samples were subjected to NGS-based targeted genetic test from July 2017 to May 2020 at the Molecular Genetics Laboratory of LHSC. Genes included in the full comprehensive panel and each targeted subpanel as per CCO guidelines are listed in **Table 1**. A total of 500 tumor samples were tested by the full comprehensive hotspot panel across a number of tumor types. A total of 1,375 NSCLC tumor samples were tested by the targeted lung subpanel, 692 metastatic colorectal tumor samples were tested for the colon subpanel, and 462 metastatic melanoma samples were subjected to targeted melanoma subpanel testing. Brain targeted subpanel testing was done for 135 glioma samples. Subpanels were tested for specific tumor types based on local funding and regulations. For example, patients with non-squamous NSCLC had reflexive testing regardless of stage, while the colorectal subpanel was run for patients with advanced disease.

**Table 1:** Genes included in the targeted panels.

Targeted panel	Genes included	Patient inclusion criteria	Number of patients tested	Number of patients positive for Tier I/II Variant
Comprehensive	ABL1, AKT1, ALK, APC, ATM, BRAF, CDH1, CDKN2A, CSF1R, CTNNB1, EGFR, ERBB2, ERBB4, EZH2, FBXW7, FGFR1, FGFR2, FGFR3, FLT3, GNA11, GNAS, GNAQ, HNF1A, HRAS, IDH1, JAK2, JAK3, IDH2, KDR, KIT, KRAS, MET, MLH1, MPL, NOTCH1, NPM1, NRAS, PDGFRA, PIK3CA, PTEN, PTPN11, RB1, RET, SMAD4, SMARCB1, SMO, SRC, STK11, TP53, VHL	Patients with advanced solid tumors; candidates for systemic therapy; ECOG performance status equal to 0 or 1 and adequate organ functions.	500	290 (58%)
Lung subpanel	ALK, BRAF, EGFR, ERBB2, KRAS, NRAS, PIK3CA, TP53	Non-small cell lung carcinoma	1,375	952 (69%)
Melanoma subpanel	BRAF, KIT, NRAS	Metastatic melanomas	462	322 (69.7%)
Colon subpanel	BRAF, KRAS, NRAS	Metastatic colorectal cancers	692	424 (61.2%)
Brain subpanel	IDH1, IDH2, BRAF	Glioma	135	28 (20.7%)

### Nucleic acid isolation

Unstained FFPE slides were reviewed and marked by a molecular pathologist to indicate the affected tumor area and cellularity for subsequent procedures. Total amount of tumor tissue used for DNA extraction was 0.25 cm^2^ or less in some cases. Tumor tissue was scraped into a 0.2-ml PCR tube. DNA purification was performed using Ion AmpliSeq™ Direct FFPE DNA Kit (ThermoFisher Scientific) according to the manufacturer’s protocol. Deparaffinization of slides was not required and only done in cases where there was little to no tissue visible on slides. In these cases, deparaffinization was done using 100% xylene followed by 100% ethanol. DNA was quantitated using the Invitrogen Qubit 3.0 Fluorometer and Invitrogen Qubit dsDNA HS Assay Kit.

### NGS library construction and sequencing

Libraries were prepared with 20 ng of genomic DNA and constructed by automated library preparation using the Ion Chef™ System and Ion Ampliseq™ Cancer Hotspot Panel v2 (Life Technologies). This panel included hotspot regions, including ~2,800 COSMIC mutations of 50 oncogenes and tumor suppressor genes with known implications in diagnosis, prognosis, and therapeutic decision-making. Regions covered and average coverage for this targeted panel are described in **Table S1**. DNA libraries of eight samples were combined into one library, which was then diluted to a concentration of 30 pmol/L. The diluted pooled DNA library was used for template preparation and chip loading on the Ion Chef system using Ion 520 chips, followed by sequencing on either the Ion PGM™ System or Ion S5™ Sequencer (ThermoFisher Scientific). Parameters used for assessing run quality included key signal >30, ISP loading >30%, and usable reads >30%. Parameters used for assessing sample quality included mean depth of coverage >1,000× and uniformity >90%. In some cases, samples were assessed at >500× mean coverage with adjusted cutoffs for variant reporting (VAF 15% with 500× coverage).

### Sequencing analysis and variant interpretation

Validation of analysis pipeline conforms to the recommendations from the Association for Molecular Pathology and the College of American Pathologists (20). We are using the Torrent Suite Software on the Torrent Server for automated sequencing data alignment and analysis. This process uses the Torrent Mapping Alignment Program (TMAP), which is specifically optimized for Ion Torrent data. Base calling, alignments, and run quality control were performed using the Ion Torrent Suite™ Software v5.8.0. Variant calling was performed by Torrent Suite™ Variant Caller plugin using standard settings. BAM and VCF files for variants were imported into Geneticist Assistant version 1.4.2 (SoftGenetics) for sample quality control assessment (minimum base coverage and mean amplicon coverage) and for databasing. As part of our analytical pipeline, we evaluate quality control by average read depth (>1,000×) and uniformity (>90%). Average coverage values for each region is listed in **Supplementary Table 1**. Beyond the quality review of each region, variant quality is also evaluated with adapted guidelines proposed by Petrackova et al. (21). Genetic variants with 5% or greater variant frequency and minimum coverage of 250 were analyzed. Variant assessments were done by genome analysts (primary review) and a clinical molecular geneticist or a molecular pathologist (secondary review). Variants were classified into four tiers (Tier I to Tier IV) based on the consensus guidelines set by the Association for Molecular Pathology, the American Society of Clinical Oncology, and the College of American Pathologists (22). Variants classified as Tier I and II were of strong clinical significance, Tier III variants were with unknown clinical significance due to lack of significant evidence, and Tier IV variants were benign or likely benign. Sequence pileups for reportable variants were manually assessed to ensure no miscalls. Variants classified as Tier I/II (described as clinically relevant variant) were reported to the oncologist/physician for each sample.

### Assay validation

NGS assay validation was performed using a retrospective set of tumor samples previously tested by other methods like allele-specific PCR with fluorescent hydrolysis probe detection (Enterogen) and sanger sequencing in our laboratory, and the limit of detection (LOD) was assessed by testing serially diluted DNA samples positive for known variants (**Supplementary File 1**).

## Results

### NGS assay validation and limit of detection

The NGS assay was clinically tested and validated by using a commercially available reference standard from Horizon Diagnostics (HDx Perkin Elmar), with known variant allele frequencies and a cohort of 39 retrospective tumor DNA samples selected from the laboratory archive, which were previously tested by other validated methods in our laboratory including allele-specific PCR with fluorescent hydrolysis probe detection (Enterogen) and sanger sequencing. These include 27 positive samples, each carrying at least one Tier I/II variant and 12 negative samples with no Tier I/II variants (**Table S1**). All the positive control samples were concordant by the NGS assay and no Tier I/II variants were detected in the negative controls. Therefore, the sensitivity and specificity of this assay for the previously assessed variants were determined to be 100%. Repeat sequencing runs were performed to assess the reproducibility and were estimated to be 100%. By testing serial dilutions of tumor DNA samples with Tier I/II variants (BRAF V600E, KRAS G12C, and EGFR exon 19 deletion p.L747_E749del), the LOD for accurate and reproducible variant calling was determined to be 5% of variant allele frequency.

### Lung subpanel assessment

**Figure 1:**
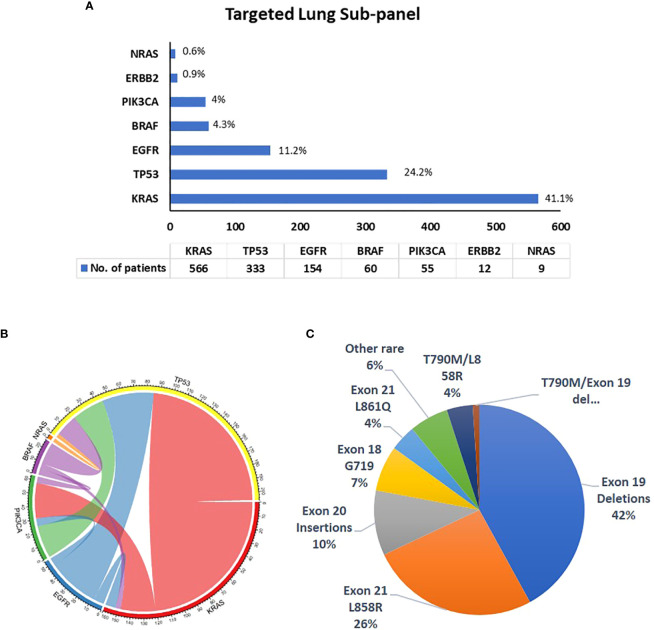
Details of variants identified by the targeted lung subpanel (*N* = 1,375). **(A)** Frequency of variants in genes identified in NSCLC tumors tested by the lung subpanel; **(B)** co-occurring mutations in NSCLC samples; **(C)** frequency of EGFR gene variants.

A total of 1,375 NSCLC samples were tested by the lung NGS subpanel, namely, 790 female samples (mean age: 69.6 years; range: 24.8 to 91.8) and 585 male samples (mean age: 69.7 years; range: 36 to 93.8 years) and 952 (69%) tested positive for reportable (Tier I or II) variants. Tier I/II variants identified are listed in **Supplementary Table 2**. The detection rates in male and female samples were 65.1% and 72.2%, respectively. Variant detection rates in specific age groups are listed in **Table 2**. KRAS was found to be the most commonly mutated gene (41.1%), followed by TP53 (24.2%) and EGFR (11.2%) (**Figure 1A**). A total of 731 samples had one variant, 205 had two variants, and 16 had three variants. Co-occurring variants are shown in **Figure 1B**. Out of 154 samples with an EGFR variant, 69 (42%) were exon 19 deletions, 42 (26%) were exon 21 L858R, 17 (10%) were exon 20 insertions, 11 (7%) were exon 18 G719S, 6 (4%) were exon 21 L861Q, 7 (4%) were T790M/L858R, 2 (1%) were T790M/Exon 19 del, and 9 (6%) were rare EGFR variants (**Figure 1C**). An interesting point noted is the high frequency of KRAS G12C mutations (15.2%; 209/1,375) in our study cohort, which was higher than the frequency of EGFR mutations (11.2%).

**Table 2:** Characteristics of NSCLC patients tested by the lung hotspot panel.

Characteristic	No. of patients	No. of patients with at least one Tier I/II Variant
Total patients	1,375	952 (69%)
Sex		
Male Female	585 790	381 (65.1%) 571 (72.2%)
Age group		
21–40 41–50 51–60 61–70 71–80 >80	8 30 178 464 507 188	7 (88%) 20 (67%) 126 (71%) 341 (73%) 346 (68%) 112 (60%)

### Colon subpanel assessment

**Figure 2:**
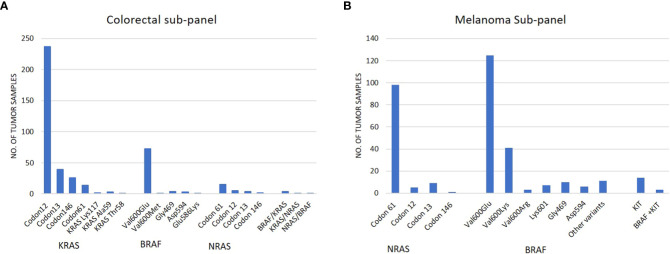
**(A)** Frequency of variants identified in tumor samples tested by the colorectal subpanel; **(B)** frequency of variants identified in tumor samples tested by the melanoma subpanel.

Out of 692 colorectal cancer samples (mean age:65.4 years; 404 male samples and 288 female samples) tested by the colon subpanel, 424 (61.2%) had a Tier I/II variant and 6 had variants in two genes (**Supplementary Table 3**). A total of 322 (46.5%) had a KRAS variant, 82 (11.8%) had a BRAF variant, and 26 (3.7%) had a NRAS variant (**Table 3**; **Figure 2A**). The most common KRAS variants were in codon 12 followed by codons 13, 146, and 61. Rare clinically relevant variants were detected in six patients. The most frequent BRAF variant was Val600Glu (V600E), which occurred in 72 samples; 10.4% of all patients had colon cancer.

**Table 3:** Gene variants in colorectal cancer samples.

Characteristic	No. of patients
Total patients Male Female Average age Range Positive for Tier I/II variant	692 404 288 65.4 years 19.8 to 90.3 years 424 (61.2%)
Variant Type	
KRAS	**317 (46%)**
BRAF	**77 (11.2%)**
NRAS	**25 (3.6%)**
BRAF/KRAS	**4**
KRAS/NRAS	**1**
NRAS/BRAF	**1**

Number of patients (% of total patients tested n=692) are written in bold.

### Melanoma subpanel

Out of 462 melanoma samples, 322 (69.7%) were positive for Tier I/II variant and 8 patients had variants in two genes (5 patients with BRAF and NRAS; 3 patients with BRAF and KIT). The highest frequency of variants was observed in the BRAF (42.2%) gene followed by NRAS (23.4%) and KIT (2.33%). The most common BRAF gene variants were in codon Val600 and NRAS codon 61 (**Table 4**; **Figure 2B**). All sample details and variants are listed in **Supplementary File 4**.

**Table 4:** Gene variants in melanoma samples.

Characteristic	No of patients
Total patients Male Female Average age Range Positive for Tier I/II variant	462 288 174 68.1 years 17.4 to 97.2 years 322 (69.7%)
Variant Type	
BRAF	195/462 (42.2%)
NRAS	108/462 (23.4%)
KIT	11/462 (2.3%)
BRAF+NRAS	5
BRAF+KIT	3

### Brain subpanel

Out of 135 brain tumor samples tested, 28 (20.7%) had one clinically relevant variant, 11 (8.1%) had a BRAF Val600Glu variant, 14 (10.3%) had an IDH1 Arg132 variant, and 3 (2.2%) had an IDH2 Arg172 variant (**Supplementary File 5**).

### Comprehensive panel

**Figure 3:**
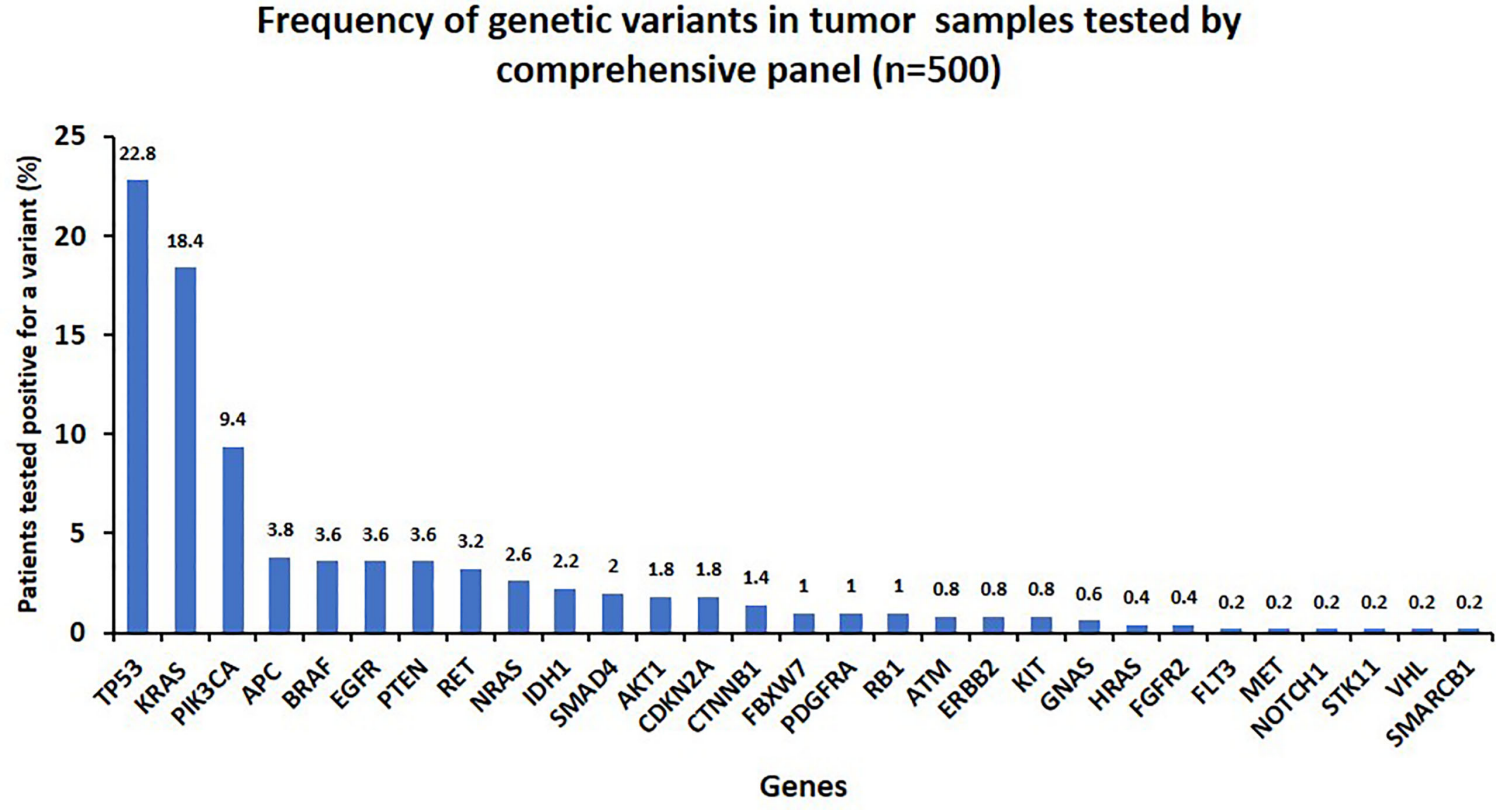
Frequency of variants in genes included in the comprehensive panel (*n* = 500).

**Figure 4:**
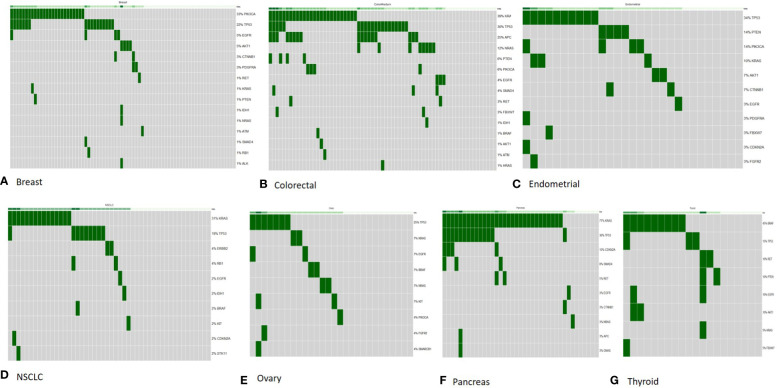
OncoPrint diagram of mutation frequencies of the genes included in the comprehensive panel in common tumor types tested in our study. All samples (with in each tumor type) are included and genes with ≥1 Tier I/II variant are shown. Each row is one gene (mutation frequencies are shown as % of total samples tested) and each column is one tumor sample. **(A)** Breast (*n* = 77); **(B)** colorectal (*n* = 67); **(C)** endometrial (*n* = 29); **(D)** non-small cell lung cancer (*n* = 48); **(E)** ovary (*n* = 28); **(F)** pancreas (*n* = 39); **(G)** thyroid (*n* = 23).

A total of 500 tumor samples from patients presenting with various cancer types were tested by a comprehensive panel with 50 genes. Two hundred ninety samples (58%) were tested positive for at least one clinically relevant Tier I/II variant. Among these samples, 82 (16.4%) had two variants and 30 (6%) had three or more variants (**Supplementary File 6**). Overall, TP53 (23%), KRAS (18.4%), and PIK3CA (9.4%) were among the most frequently mutated genes, followed by APC, BRAF, PEN, RET, NRAS, IDH1, SMAD4, AKT1, and CDKN2A with a detection rate ranging from 1.4% to 3.8% (**Figure 3**; **Supplementary File 6**). Other less frequently mutated genes were FBXW7, PDGFRA, RB1, ATM, ERBB2, KIT, GNAS, HRAS, FGFR2, Flt3, MET, NOTCH1, STK11, VHL, and SMARCB1 with ≤1% detection rate (**Table 5**). Oncoprint plots (**Figure 4**) shows overview of alterations in each sample with in common tumor types analyzed in this study.

**Table 5:** Frequency of genetic variants in tumor types tested by the comprehensive gene panel.

Tumor type	No. of patients tested	No. of patients with Tier I/II variant (%)	Genes with ≥4 patients with a Tier I/II variant (no. of patients; %)	Genes with ≤3 patients with a Tier I/II variant
Breast	77	45 (58.4%)	PIK3CA (25; 32.4%) TP53 (17; 22%) EGFR (4; 5.2%)	CTNNB1, AKT1, EGFR, PDGFRA, PTEN, ATM, RB1, KRAS, NRAS
Colorectal	67	52 (77.6%)	KRAS (26; 38.8%) TP53 (20; 29.8%) APC (17; 25.3%) NRAS (8; 11.9%) PIK3CA (4; 5.9%)	SMAD4, FBXW7, PTEN, EGFR
NSCLC	48	29 (60.4%)	KRAS (15; 31.2%) TP53 (9; 18.7%)	EGFR, ERBB2, RB1, BRAF, CDKN2A, IDH1, STK11, KIT
Pancreas	39	33 (84.6%)	KRAS (28; 71.7%) TP53 (14; 35.8%) CDKN2A (4; 10.2%)	NRAS, EGFR, SMAD4, CTNNB1, APC, GNAS, RET
Endometrial	29	21 (72.4%)	TP53 (10; 34.4%) PTEN (4; 13.7%) PIK3CA (4; 13.7%)	PTEN, CTNNB1, KRAS
Ovary	28	16 (57.1%)	TP53 (7; 25%)	BRAF, KRAS, NRAS, FGFR2, EGFR, PIK3CA, SMARCB1, KIT
Thyroid	23	17 (73.9%)	BRAF (9; 39.1%) RET (4; 17.3%)	TP53, AKT1, EGFR, FBXW7, ATM, PTEN, KRAS
Unknown primary	17	10 (58.8%)	TP53 (4; 23.5%)	MET, BRAF, KRAS, RB1, IDH1, KIT, PIK3CA, TP53
GE junction	13	6 (46.2%)	TP53 (6; 46.1%)	
Renal	13	3 (23.1%)		PTEN, VHL, TP53
Cholangiocarcinoma	11	4 (36.4%)		IDH1, RET, EGFR, CTNNB1, KRAS, TP53, SMAD4, PIK3CA
Esophagus	11	4 (36.4%)	TP53 (4; 36.3%)	PIK3CA, CTNNB1
Bladder	10	5 (50%)		TP53, KRAS, SMAD4, PIK3CA, CTNNB1, GNAS
Cervix	10	3 (30%)		KRAS, PIK3CA, SMAD4
Oral	10	2 (2%)		TP53, NOTCH1; PIK3CA
Gastric	9	4 (44.4%)		FBXW7, TP53, GNAS, KRAS, ERBB2
Sarcoma	9	3 (33.3%)		IDH1, TP53
Adenoid cystic carcinoma	8	2 (25%)		PTEN, CTNNB1, PDGFRA
Melanoma	7	5 (71.4%)		EGFR, TP53, NRAS, RB1, CDKN2A, BRAF
Parotid	6	3 (50%)		ERBB2, FLT3, PIK3CA, HRAS
Prostate	6	1(16.7%)		PTEN

*Breast:* Out of 77 breast tumor samples, 45 (58.4%) had a Tier I/II variant with a high frequency of variants observed in PIK3CA (32.4%) and TP53 (22%) genes. Variants in more than one gene were detected in 13 patients and 7 of these patients had variants in both TP53 and PIK3CA gene (**Figure 4**).

*Colorectal:* Out of 67 colorectal tumor samples, 52 (77.6%) tested positive and the most frequently mutated genes included KRAS (38.8%), TP53 (29.8%), and APC (25.35) followed by NRAS (11.9%) and PIK3CA (5.9%). Co-occurrence of variants in more than one gene was common in colorectal cancer samples as 32 patients tested positive for variant in more than one gene. The most common co-occurrence of variants was observed in KRAS, APC, and TP53 genes (**Figure 4**).

*Non-small cell lung cancer*: Forty-eight NSCLC tumor samples tested by the comprehensive panel also showed high frequency of variants in KRAS (31.2%) and TP53 (18.7%) genes, which was in line with the targeted lung subpanel.

*Pancreatic:* Approximately 33 of 39 pancreatic tumor samples had a clinically relevant variant with KRAS (71.7%), TP53 (35.8%), and CDKN2A (10.2%) observed as the most common genes with a variant. A total of 13 pancreatic tumor samples had co-occurring variants in KRAS and TP53 genes (**Figure 4**).

*Endometrial:* Out of 29 endometrial cancer samples, 21 (72.4%) tested positive and 34.4% had variants in TP53 gene, and 13.7% in PTEN and PIK3CA gene each.

*Ovarian:* Out of 28 ovarian cancer samples, 16 (57.1%) tested positive and 25% had variants in TP53 gene.

*Thyroid tumor:* Out of 23 thyroid tumor samples, 17 (73.9%) had genetic variants. BRAF gene was the most commonly mutated, in 39% of patients, followed by RET gene in 17.3% of patients.

*Rare tumors:* Some of the less frequent advanced tumors tested by the comprehensive panel in our study cohort include renal, esophagus, bladder, cervix, oral, gastric, sarcoma, melanoma, and prostate. The frequencies of variants in genes observed in these tumors are listed in **Table 5**.

## Discussion

Molecular profiling of solid tumors is rapidly becoming the standard of care for a widening range of oncology indications with strong implications in diagnosis, prognostic outcomes, and clinical and treatment management decisions. NGS-based targeted hotspot panel assays provide a cost-effective platform for rapid and accurate molecular characterization of solid tumors in a clinical laboratory setting. We first validated a targeted NGS cancer hotspot panel assay and then implemented it to identify genomic aberrations in patient tumor samples. In the present study, we have analyzed the frequency and nature of variants in 3,164 solid tumor samples from patients with advanced cancer received in our laboratory for genetic testing using this assay. This study provides real-world evidence for the utility of hotspot genotyping and small targeted tumor-specific panel sequencing.

### Diagnostic yields of target subpanels *vs*. comprehensive panels

Target subpanels are designed to test specific genes and/or variants with known clinical utility for the tumor type. These genes are recommended by clinical guidelines and have a direct impact on patient management. Target panels have the advantage of streamlining data analysis and interpretation, expediting the results, and decreasing the rate of variants of unknown clinical significance. In our setting, NGS panel analysis resulted in high diagnostic yields across various clinical indications including lung (69%), colon (61.2%), melanoma (69.7%), and brain (20.7%), highlighting the utility of targeted subpanels in the clinical setting. In contrast, comprehensive panels have the ability to identify genes known to be targetable in other cancer and other genes with therapeutic or prognostic potential and/or diagnostic markers that may be incorporated in the clinical practice as more evidence become available. For example, comparison of data from colorectal tumor showed a significant diagnostic yield in genes such as TP53 and APC that are currently not part of the targeted panel. Overall, the diagnostic yield of the comprehensive panel was 58%, ranging from 77.6% in colorectal cancer to 2% in oral cancer (**Table 5**).

### Frequency of variants in targeted panels

Genetic mutations observed in NSCLC may vary among patients with different racial and demographic background and other clinical and pathological aspects (23, 24). However, we do not have the exact ethnicity information available for patients included in our study but overall frequency of variants in NSCLC observed in our study is in accordance with other reported studies from the Western population. For example, EGFR variants seen in 11% of our samples are reported in literature at nearly 15% among North Americans, in contrast to nearly 50% in Asians (23). The most common EGFR mutations are either exon 19 deletions or exon 21 L858R found at 42% and 26%, respectively, in our dataset, which is comparable but slightly lower than what has been reported in literature: exon 19 deletions at 42.8% and exon 21 L858R substitutions at 29.8% reported in a metanalysis of studies from many regions including North America (23). In our population, the frequency of less common EGFR mutations, exon 20 insertions (10%), G719X (7%), and L861Q (4%), are all higher than expected (25).

Similarly, KRAS mutation seen at 41.1% in our study is reported to range from one-fourth to one-third (∼15%–35%) of the NSCLC patients in the Western population to 4%–8% in Asians (26, 27). In our dataset, patients had KRAS G12C mutations in 15.2% of samples. This is higher than our rate of EGFR positivity, and nearly double the frequency of the common EGFR mutations (exon 19 deletions and L858R) combined.

BRAF and KRAS mutations have been reported to occur in ∼10% and 44% of patients with metastatic colorectal cancer, which is similar to frequency (KRAS: 46%; BRAF: 11.2%; NRAS: 3%) observed in our sample cohort (28). Frequency of variants identified in melanoma samples as listed in **Table 4** is in line with reported studies (BRAF ∼50%, NRAS ∼25%; KIT ∼4.5%), as analyzed by Vanni et al. from different reported studies (29, 30). Glioma samples tested by the brain subpanel had BRAF variants at 8% and IDH1/IDH2 variants at 12.5%. As reported earlier, we noted a higher incidence of BRAF variants in younger patients (the median age of patients with a BRAF variant is 31.5 years *vs*. the median age of patients without a BRAF variants is 56.6 years) (31). However, it was noted that the overall frequency of variants across different tumor types identified by our targeted subpanels is comparable to reported literature but detailed analysis about the frequency of specific variant types based on precise tumor histology and clinical and demographic details is beyond the scope of this study as this information was not available in our laboratory-based database.

### Co-occurring variants in targeted subpanels

Out of 1,375 NSCLC samples tested by the lung subpanel, 69% (952) tested positive for a variant. Co-occurrence of mutation in more than one gene was observed in 260/1,375 (19%) patients. Co-occurring variants in oncogenic drivers and tumor suppressors in NSCLC tumors contribute towards complex molecular diversity of these tumors and may impact the efficacy of TKIs. The most common co-occurring genetic alteration observed in our study include TP53 (207 samples) with 121 KRAS, 40 EGFR, 25 PIK3CA, 17 BRAF, 3 NRAS, and 1 ERBB2 positive samples. Concurrent TP53 mutations with KRAS and EGFR are reported as negative prognostic markers for advanced NSCLC patients who are candidates for EGFR-TKI or ALK-TKI treatments (32). Dual mutations in the EGFR gene like T790M/Exon 19 del and T790M/L858R were observed and are known to alter the response to TKI therapy (33). We also observed tumor samples with co-occurring driver variants in EGFR, KRAS, and BRAF genes (seven patients with KRAS/EGFR variants; four patients with BRAF/KRAS; one patient with BRAF/EGFR/KRAS). Typically considered as mutually exclusive, concurrent driver mutations in EGFR/KRAS/BRAF genes are rare in literature (34, 35) and their clinical relevance is not well known but it has been suggested that patients with co-occurring actionable variants may require tailoring the combination or sequential treatment strategies (36). Among the samples tested by the colon subpanel, a high frequency of mutations was noted in KRAS (46.5%) and BRAF (11.8%) genes, and four patients had concomitant KRAS and BRAF (nonV600E) variants. BRAF and KRAS are two key oncogenes that determine response to anti-EGFR therapies in colorectal cancer patients. BRAF and KRAS variants are mutually exclusive and co-occurrence is rarely reported (37, 38). However, clinical outcomes for such patients is not well characterized but personalized combination therapeutic strategies have been suggested for such patients (28). Also, the co-occurrence of NRAS and BRAF, the two most frequently mutated genes in melanoma, is also rarely reported (2.9%), which is in agreement with our observations as we found concomitant NRAS/BRAF variants in 5 (1%) of our melanoma samples (39); however, their clinical impact is not known.

### Comprehensive panel testing

A growing number of genomic aberrations are impacting the treatment decisions for advanced metastatic tumors. We implemented a comprehensive NGS assay covering 50 oncogenes and tumor suppressor genes to identify variants in genes with a potential clinical impact in a variety of tumor types, including breast, colorectal, pancreas, endometrial, ovary, lung, and thyroid tumors. To evaluate the molecular profile, 500 tumor samples were assessed by this assay and a positive mutation rate of 58% was observed. Some of the frequently mutated genes identified in these tumors hold high clinical relevance.

Sequencing studies analyzing somatic driver mutations in genetically complex tumors like breast cancer have identified PIK3CA as a frequently mutated gene in breast tumors followed by TP53 with co-occurrence reported in 10%–15% patients (40, 41). We detected variants in PIK3CA at 33%, TP53 at 22%, and co-occurring variants in 9% patients (7/77). Variants in other clinically relevant genes were less frequent with EGFR and AKT1 variants found in 5% patients. The PI3-kinase inhibitor in combination with the estrogen receptor (ER) antagonist is FDA-approved for the treatment of patients with PIK3CA mutant ER+/HER2− breast cancer and the presence of PIK3CA/TP53 mutations has evidence of prognostic relevance (42, 43).

Colorectal tumors had the highest frequency of variants (77.6%) as compared to other tumors analyzed in our study. In line with previous studies, colorectal cancer samples had high frequency of variants in genes including KRAS, TP53, APC, NRAS, PTEN, and PIK3CA. Other than RAS genes that predict anti-EGFR sensitivity, PIK3CA mutations have prognostic implication in colorectal tumors. PIK3ACA mutations are associated with a worse response to first-line chemotherapy (11) and a phase I clinical trial has shown evidence for the sensitivity of PIK3CA mutated colorectal cancer to the PIK3a-selective inhibitor (44). Early trial data suggest that PI3K inhibitors may be of benefit to solid tumors harboring PTEN mutation (45). There is evidence that co-occurring variants in APC, PIK3CA, TP53, and KRAS may impact the overall disease progression and response but it is not yet well studied and larger trials are needed to fully understand the impact on disease outcome (46).

Following TP53 (34.4%), PTEN and PIK3CA were the most commonly mutated genes in endometrial tumors, and these variants have predictive value in endometrial tumors. PTEN has been associated with a favorable outcome in endometrial cancer, and pre-clinical data have shown that inactivating mutations in the PTEN gene may confer sensitivity to PI3K-AKT inhibitors as well as PI3K/mTOR inhibitors (14, 45). Since PIK3CA is another frequently mutated gene in endometrial tumors, PIK3CA-directed inhibitors may show benefits but its utility is still under investigation (47). Recurrent TP53 mutations in endometrial tumors have been associated with higher rates of recurrence in grade 1–2, stage I and II endometrioid adenocarcinoma (48, 49). CTNNB1 gene variants were less frequent at 7% and are associated with increased risk of recurrence in grade 1–2 early-stage endometrial endometrioid adenocarcinoma (50).

KRAS gene alterations are the most frequent, observed at a frequency of 72% in pancreatic tumor samples, with TP53 at 36% as the second most common followed by CDKN2A. The high prevalence of KRAS variants in pancreatic tumors suggests the clinical utility of therapies targeting the RAS signaling pathway, but studies done in this direction so far have shown little clinical benefit (51). However, KRAS mutations have been reported to have a negative impact on prognosis and improve the performance of classic cytopathology to diagnose pancreatic tumors (52).

Forty-eight NSCLC tumor samples tested by the comprehensive panel had KRAS (31.2%) and TP53 (18.7%) as the most frequently mutated, followed by ERBB2, RB1, and EGFR. EGFR mutations predict response to anti-EGFR therapies in NSCLC tumors, and TP53 mutations have been reported to have a negative prognostic effect (6). Oncogenic variants in ERBB2 gene may confer sensitivity to anti-HER2-directed therapies and trastuzumab deruxtecan is now FDA-approved for these patients (53–55).

Thyroid cancer is another tumor type with a high mutation positivity rate of 74% with BRAF at 40% followed by RET at 17.3%. BRAF positive thyroid tumors have shown sensitivity to RAF and/or MEK inhibitors (56) and RET variants correlate with aggressive phenotype and worse outcome (57). Co-occurring variants also predict survival in thyroid tumors (58, 59).

### Disease-specific subpanels/comprehensive panel in the clinical setting

A molecular testing model implemented in Ontario as per CCO guidelines represents the integration of both comprehensive and targeted subpanels for tumor samples in the clinical setting. The data collected here are not sufficient to make any conclusions about the utility of one over the other, but this model definitely highlights the feasibility of administering both in a clinical environment with evidence of high variant detection rates. We have targeted ongoing studies to establish the utility of subpanel *vs*. full panel testing and also to investigate disease-specific clinical scenarios suited best for each type of testing.

## Conclusion

NGS has revolutionized the way cancer is diagnosed and treated. This study demonstrated the utility of NGS in identifying actionable genetic alterations in solid tumors, including the potential for identifying novel therapeutic targets. While the study had some limitations, such as targeting specific hotspot regions and lack of additional clinical information, the results showed a high diagnostic yield of 58% using a comprehensive panel and 20.7%–69.7% using targeted subpanel testing. The study also highlighted the efficiency of incorporating both comprehensive and targeted subpanel testing in a clinical laboratory setting. Additionally, the study identified co-occurring driver mutations and novel gene mutations, emphasizing the need for continued research to expand precision medicine to all tumor types. Overall, this study provides promising evidence for the utility of NGS testing in clinical laboratories for diagnosing and treating solid tumors.

## Data availability statement

The data analyzed in this study is subject to the following ethical restrictions: Raw NGS files cannot be made available due to patient confidentiality and institutional requirements. Specifically, these data are from patient samples within the clinical testing laboratory. The samples were obtained through clinical testing requisitions. Samples are blinded and assessed through our clinical laboratory Quality Improvement Protocol as part of the clinical test/technology validation. Requests to access these datasets should be directed to the corresponding author.

## Ethics statement

Ethical review and approval was not required for the study on human participants in accordance with the local legislation and institutional requirements. Written informed consent to participate in this study was provided by the participants’ legal guardian/next of kin.

## Author contributions

PB and JT analyzed and interpreted the data, wrote the manuscript. HL, BS planned and executed this study and contributed in reviewing the manuscript. DB, CH, SW, MC, LP contributed in patient enrollment and analyzing the clinical impact. JK, AS, HL, SS, ML and BS validated the NGS assay and contributed in clinical implementation of the NGS assay. AF, AS ML, HL, LP, BS and JK contributed in data collection and analysis. PB, JT, HL, BS, MC, CH analyzed the NGS findings and performed clinical interpretations. All authors contributed to the article and approved the submitted version.
